# Associations of Cardiorespiratory Fitness and Body Mass Index with Incident Restrictive Spirometry Pattern

**DOI:** 10.1136/bjsports-2022-106136

**Published:** 2023-01-06

**Authors:** Joey M. Saavedra, Angelique G. Brellenthin, Bong Kil Song, Duck-chul Lee, Xuemei Sui, Steven N. Blair

**Affiliations:** 1 Iowa State University, Department of Kinesiology, Ames, IA 50014, USA; 2 University of South Carolina, Department of Exercise Science, Arnold School of Public Health, Columbia, SC 29208, USA

**Keywords:** Cardiorespiratory fitness, obesity, lung function, restrictive spirometry, Aerobics Center Longitudinal Study

## Abstract

**Objectives:**

Restrictive spirometry pattern suggests an impairment of lung function associated with a significantly increased risk of premature mortality. We evaluated the independent and joint associations of cardiorespiratory fitness and body mass index with incident restrictive spirometry pattern.

**Methods:**

Data from the Aerobics Center Longitudinal Study included 12,360 participants (18–82 years). CRF was assessed by maximal treadmill test and categorized into five groups. Body mass index was categorized into normal weight (<25.0 kg/m^2^), overweight (25.0–29.9 kg/m^2^), or obesity (≥30.0 kg/m^2^). Restrictive spirometry pattern was defined as the simultaneous occurrence of forced expiratory volume in 1-second/force vital capacity ≥ lower limit of normal and forced vital capacity < lower limit of normal.

**Results:**

There were 900 (7.3%) cases of restrictive spirometry pattern (mean follow-up: 6.9 years). Compared to ‘category 1 (least fit),’ hazard ratios (HRs) (95% confidence intervals *CIs+) of RSP were 0.78 (0.63–0.96), 0.68 (0.54–0.86), 0.70 (0.55–0.88), and 0.59 (0.45–0.77) in categories 2, 3, 4, and 5 (most fit), respectively, after adjusting for confounders including body mass index. Compared to normal weight, HRs (95% CIs) of RSP were 1.06 (0.91–1.23) and 1.30 (1.03–1.64) in overweight and obese, respectively. However, the association between obesity and restrictive spirometry pattern was attenuated when additionally adjusting for cardiorespiratory fitness (HR: 1.08, 95% CI: 0.84–1.39). Compared to the ‘unfit and overweight/obese’ group, HRs (95% CIs) for restrictive spirometry pattern were 1.35 (0.98–1.85), 0.77 (0.63–0.96), and 0.70 (0.56–0.87) in the ‘unfit and normal weight,’ ‘fit and overweight/obese,’ and ‘fit and normal weight’ groups, respectively.

**Conclusions:**

Low cardiorespiratory fitness was associated with a greater incidence of restrictive spirometry pattern, irrespective of body mass index. Future studies are needed to explore potential underlying mechanisms of this association and to prospectively evaluate if improving cardiorespiratory fitness reduces the risk of developing restrictive spirometry pattern.

## INTRODUCTION

Restrictive spirometry pattern (RSP) is an impairment of lung function with a prevalence of 5–10% in the United States and Europe.[1, 2] RSP is assessed through spirometry: a non-invasive evaluation of the flow rate and volume of forcefully expired air from the maximally inflated lung.[3] Primary indices of spirometry include the forced expiratory volume in 1-second (FEV_1_), the forced vital capacity (FVC), and the ratio between the two (FEV_1_/FVC). A ratio ≥0.70 is broadly indicative of optimal airway functioning.[4] However, when a normal ratio is accompanied by an abnormally low FVC, the result is RSP.[2] Compared to those with entirely normal spirometry, individuals with RSP have a two-fold increase in the risk of premature mortality.[5] Strategies to reduce the occurrence of RSP could therefore have far-reaching implications for longevity in the general population.

A potent risk factor of RSP is obesity,[6] defined as a body mass index (BMI) ≥30 kg/m^2^,[7] which affects 42% and 28% of adults in the USA and England, respectively.[8, 9] Obesity contributes to RSP by limiting lung and chest wall compliance,[6] damaging the elastic properties of lung tissue through inflammation,[10] and hampering the ability of the lung to properly inflate. Current projections suggest that 48% of the American and 36% of British population will be obese by the year 2030 and 2040, respectively.[11, 12] Even modest annualized gains in body mass (>0.25 kg/year) can accelerate the decline in lung capacity,[13] exacerbating the long-term risk of RSP. Therefore, the prevalence of RSP and its associated mortality risk will likely worsen with time.

High cardiorespiratory fitness (CRF) has been shown to negate the associations of obesity with several health outcomes including premature mortality, resulting in a ‘fat-but-fit’ phenotype that favors health and longevity relative to ‘unfit’ phenotypes of any weight (including normal weight).[14] Additionally, high CRF is associated with better pulmonary function and lung health in general.[15, 16] CRF is a reflection of the body’s integrated ability to transport and utilize oxygen and is considered a clinical vital sign due to its strong and consistent associations with reduced mortality risk.[17] High CRF may counteract the obesity-driven etiology of RSP although there are limited data.

We therefore examined the independent and combined associations of CRF and BMI with incident RSP among adults. We hypothesized that 1) higher baseline CRF would be independently associated with a lower risk of RSP at follow-up, and 2) higher baseline CRF would attenuate the risk of incident RSP among those with overweight or obesity at baseline.

## METHODS

### Study Design and Sample

The Aerobics Center Longitudinal Study (ACLS) is a prospective study of men and women who attended the Cooper Clinic for preventive medical examinations in Dallas, TX. Participants were encouraged to attend the Cooper Clinic either by their employers or by their primary care physicians, although some individuals were self-referred after hearing about the study from others. Therefore, ACLS recruitment was not generally representative of the population at large, and follow-up intervals varied across participants. ACLS participants are community-dwelling, predominantly non-Hispanic white (>95%), college educated, from middle-to-upper socioeconomic strata, and generally healthy at baseline.[18] Baseline examinations included anthropometric measurements, 12-hour fasting blood chemistry, medical and lifestyle history questionnaires, pulmonary function measures (spirometry), and a maximal treadmill test. We included men and women who had at least two medical examinations between 1974 and 2003 to assess the associations of baseline CRF and BMI on the incidence of RSP at follow-up. Participants provided written informed consent, and the study underwent annual review and approval by the institutional review board of The Cooper Institute.

We began with a sample of 14,295 participants free from RSP at baseline and who had complete data on CRF, BMI, spirometry, and other covariates. We then excluded individuals with asthma (*n*=1,078), COPD (*n =*395), cancer (*n*=341), and CVD including stroke and myocardial infarction (*n*=121) to minimize the potential effects of confounding, resulting in a final analytical sample of 12,360 men and women aged 18–82 years.

### Measurement of Cardiorespiratory Fitness and Body Mass Index

CRF was determined by maximal treadmill test to volitional exhaustion using a modified Balke protocol,[19] which is described in depth elsewhere.[20] The final treadmill speed and grade were used to estimate CRF in metabolic equivalents (METs) using the following equation from the American College of Sports Medicine: [3.5 + (0.1 * speed) + (1.8 * speed * grade)] / 3.5. We excluded individuals who failed to reach 85% of their age-predicted maximal heart rate to limit potential confounding caused by an underestimation of CRF. We created CRF categories based on the sex- and age-specific categories of CRF distribution of the entire ACLS cohort, as done in previous ACLS studies.[21, 22] This approach was taken because of the absence of standardized cut-points for low CRF. Additionally, this approach accounts for the unique characteristics of the ACLS (i.e., participants are predominantly white, male, and from higher socioeconomic strata). The first category of CRF was defined as the ‘least fit’ (referent), followed by categories 2, 3, 4, and 5 (most fit). BMI was calculated as measured body weight in kilograms (kg) divided by height in meters squared (m^2^), and participants were classified as either normal weight (<25.0 kg/m^2^), overweight (25.0–29.9 kg/m^2^), or obese (≥30.0 kg/m^2^) using World Health Organization (WHO) cut-points.[7] For the joint analysis of CRF and BMI on incident RSP, we dichotomized CRF into ‘unfit’ and ‘fit’ categories (lowest 20% and highest 80% of the ACLS CRF distribution, respectively) based on previous ACLS studies.[23, 24] We also combined the overweight and obese phenotypes into a single group, resulting in two BMI categories overall (normal weight and overweight/obese). We then created four CRF/BMI combinations to permit an assessment of the joint associations of CRF and BMI on incident RSP: ‘unfit and overweight or obese’ (referent), ‘unfit and normal weight’, ‘fit and overweight or obese’, and ‘fit and normal weight’.

### Measurement of Lung Function

Pre-bronchodilator lung function was assessed by trained technicians using a Collins 421 Survey spirometer. The primary indices of spirometry were the forced expiratory volume in 1-second (FEV_1_), the forced vital capacity (FVC), and the ratio between the two (FEV_1_/FVC). Each participant completed at least three forced expirations, and the data from the single best trial (i.e., the highest expiratory maneuver) were used to characterize lung function. We defined RSP as FVC <lower limit of normal (LLN), a commonly used cut-point that is equal to the 5^th^ percentile of a healthy, non-smoking population,[25] coupled with FEV1/FVC ≥LLN, following the approach adopted by previous studies.[2, 26, 27] The present study utilized the Global Lung Initiative (GLI) reference population to establish the LLN for all spirometric indices.[28] Participants who developed RSP within one year of follow-up were excluded to minimize the potential for reverse causality.

Given the wide range of participant ages in the present study (18–82 years), we chose to characterize RSP using the LLN rather than the fixed threshold approach (FVC < 80% of predicted and FEV1/FVC > 0.7) to reduce the risk of misclassification.[29] There is growing consensus that fixed thresholds for the classification of spirometric impairment result in increased rates of false negative classification in younger participants, and increased rates of false positive classification in older participants.[30] Therefore, LLNs derived from the GLI prediction equations provide an international diagnostic standard that is less prone to age-related bias.[4]

### Assessment of Covariates

Covariates were obtained from the medical and lifestyle history questionnaire given at baseline, and included age (years), sex (male or female), smoking status (never, former, of current), examination year, heavy alcohol intake (>7 drinks/week for women or >14 drinks/week for men),[31] meeting the aerobic physical activity guidelines (≥500 MET-min/week of aerobic physical activity), diabetes (defined as self-reported use of insulin, a physician-diagnosed history of diabetes, or fasting plasma glucose concentration ≥126 mg/dL), hypertension (blood pressure ≥130/80 mmHg or a physician diagnosis of hypertension), and baseline FVC (litres).

### Statistical Analysis

Baseline differences between RSP cases and non-cases were assessed using χ^2^ for categorical variables and general linear models for continuous variables. We used Cox proportional hazard models, adjusting for potential confounders, to estimate hazard ratios (HRs) and 95% confidence intervals (95% CIs) of incident RSP using three main approaches: 1) categories of CRF as the main exposure, 2) categories of BMI as the main exposure, and 3) the four combined categories of CRF/BMI as the main exposures (joint analyses). Cumulative hazard plots grouped by CRF or BMI showed no significant violations of the proportional hazard assumption. We evaluated effect modification by performing stratified analyses according to sex, age (<45 or ≥45 years), BMI (normal weight, overweight, obese), smoking status (never, former, current), heavy alcohol intake (yes or no), meeting aerobic physical activity guidelines (yes or no), diabetes (yes or no), and hypertension (yes or no). To further assess the robustness of our findings, we redefined obesity in a subset of 8,685 participants with available waist circumference (WC) data. We used cut-points adopted by the National Heart, Lung, and Blood Institute (NHLBI) to define abdominal obesity (high WC) in this subset: >102 cm or >88 cm for men or women, respectively.[32] Using the same CRF cut-points for the CRF/BMI joint analyses, we additionally created four CRF/WC combinations to assess the joint associations of these two exposures on incident RSP: ‘fit and normal WC’, ‘fit and high WC’, ‘unfit and normal WC’, ‘unfit and high WC’. Additional sensitivity analyses were performed to assess associations of CRF and BMI on RSP when RSP was defined as Preserved Ratio Impaired Spirometry (PRISm); [33] when ‘unfit’ was defined as the lower third of CRF in this sample (rather than the bottom 20% of the ACLS distribution); and when overweight and obesity were maintained as separate categories (rather than combining the two). All data were analyzed using SAS version 9.4 (SAS Institute Inc.), and we considered a 2-sided *P*-value < 0.05 to be significant.

## RESULTS

There were 900 cases (7.3%) of RSP over an average follow-up time of 6.9 years. At baseline, those who developed RSP had higher BMIs, were more likely to be current smokers, were less ‘fit’, were less likely to meet the aerobic physical activity guidelines, had a higher prevalence of diabetes or hypertension, and had lower indices of pulmonary function (FEV_1_ and FVC) (Table 1). Baseline characteristics of the sample by categories of CRF and categories of BMI can be found in the online supplemental materials (Tables S1 and S2, respectively).

Compared to the first category of CRF (least fit), the HRs (95% CIs) for incident RSP were 0.78 (0.63–0.96), 0.68 (0.54–0.86), 0.70 (0.55–0.88), and 0.59 (0.45–0.77) for the second, third, fourth, and fifth categories of CRF, respectively (*P* for linear trend <0.001), after adjusting for potential confounders including BMI (Table 2). Compared to normal weight, HRs (95% CIs) of RSP were 1.06 (0.91–1.23) and 1.30 (1.03–1.64) respectively, after adjusting for potential confounders except CRF (Model 2). However, after further adjusting for CRF, the fully adjusted HRs (95% CIs) for incident RSP were 0.98 (0.84–1.14) and 1.08 (0.84–1.39) in the overweight and obesity categories, respectively (Table 2).

**Figure 1:**
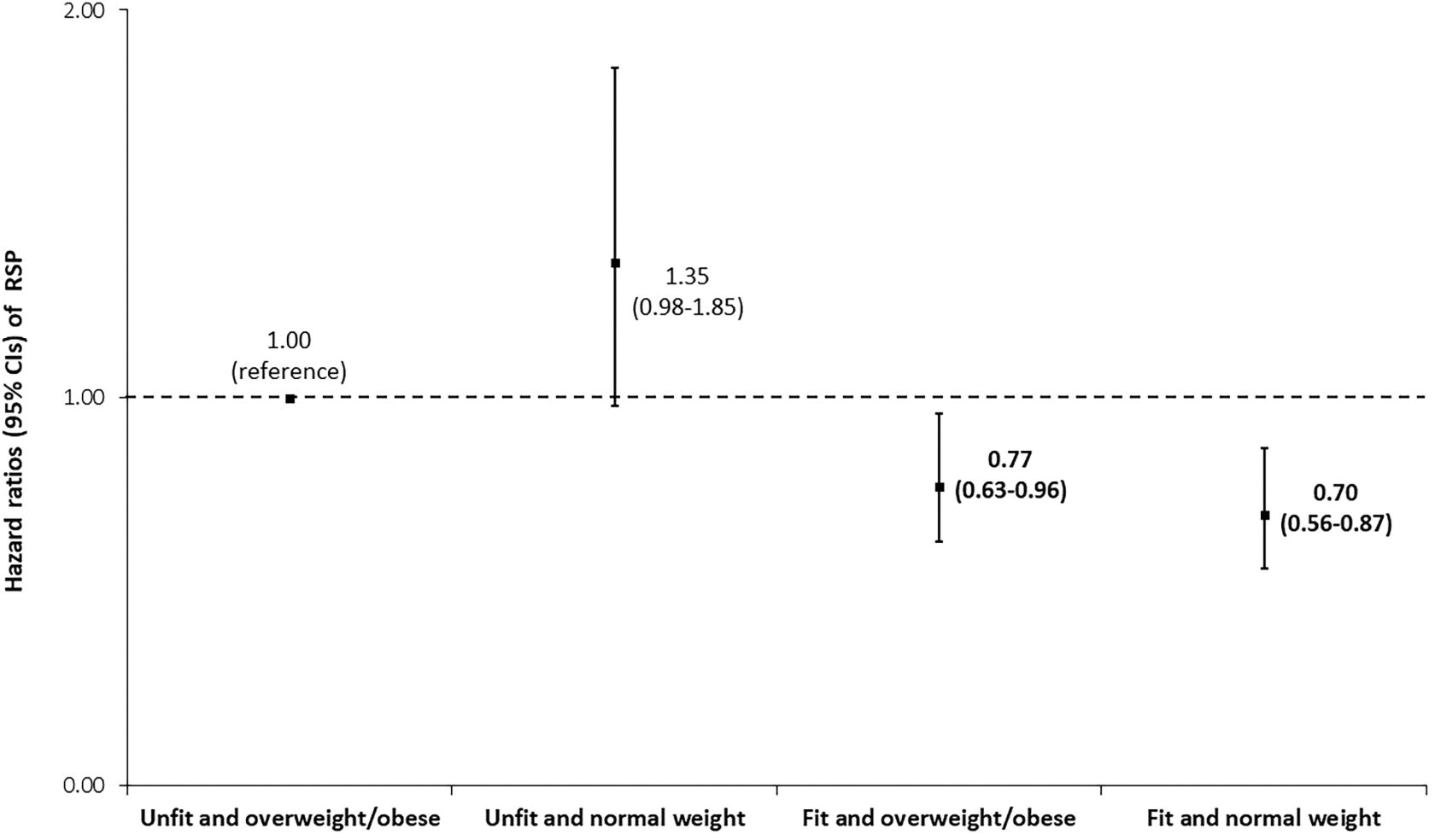
Joint associations of cardiorespiratory fitness (CRF) and body mass index (BMI) with incident restrictive spirometry pattern. Participants were divided into four groups based on combined categories of cardiorespiratory fitness (‘unfit’ or ‘fit’) and body mass index (normal weight or overweight/obesity). ‘Unfit’ was defined as the bottom 20% of the cardiorespiratory fitness distribution, and ‘fit’ was defined as the upper 80%. Normal weight was defined as body mass index <25.0 kg/m^2^, while overweight or obese was defined as was ≥25 kg/m^2^. The cox proportion hazard model was adjusted for sex, age (years), examination year, smoking status (never, former, current), heavy alcohol intake (yes or no), meeting the aerobic physical activity guidelines (yes or no), diabetes (yes or no), hypertension (yes or no), and baseline FVC (L). The number of participants (and cases of restrictive spirometry pattern) in the ‘unfit and overweight/obese’, ‘unfit and normal weight’, ‘fit and overweight/obese’, and ‘fit and normal weight’ categories were 959 (128), 339 (56), 5282 (342) and 5780 (374) respectively. Bolded values indicate statistically significant difference from the reference group (*P* < 0.05).

**Figure 2:**
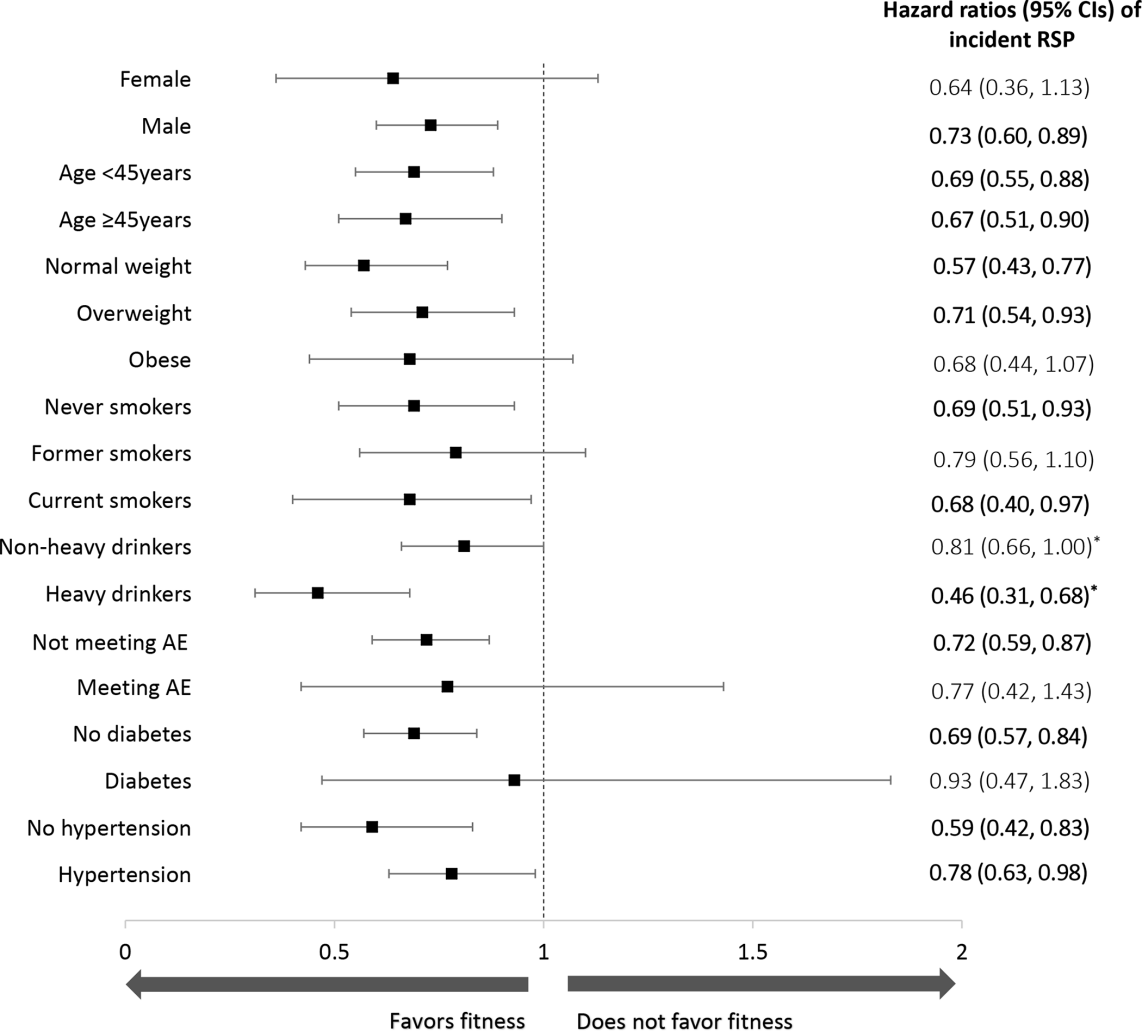
Hazard ratios (95% CI) of incident restrictive spirometry pattern (RSP) based on cardiorespiratory fitness (CRF) among subgroups. Hazard ratios (HRs) are depicted by the black squares and 95% confidence intervals by whiskers. The reference group for all analyses was the ‘unfit’ group (bottom 20% of the CRF distribution). The model was adjusted for examination year, sex (not in sex-stratified analysis), age (not in age-stratified analysis), BMI category (not in BMI-stratified analysis), current smoking status (not in smoking-stratified analysis), heavy alcohol drinking (not in drinking-stratified analysis), meeting aerobic exercise (AE) guidelines (not in AE-guideline-stratified analysis), diabetes (not in diabetes-stratified analysis), and hypertension (not in hypertension-stratified analysis). The *P* value for interaction was >0.05 for all stratified analyses except for heavy drinking (*), where *P* =.032.

In a joint analysis (Figure 1), compared to the ‘unfit and overweight/obese’ group, the adjusted HRs (95% CIs) of incident RSP were 1.35 (0.98–1.85), 0.77 (0.63–0.96) and 0.70 (0.56–0.87) for the ‘unfit and normal weight’, ‘fit and overweight/obese’, and ‘fit and normal weight’ groups, respectively. Higher CRF was also associated with consistently reduced risks of RSP (all HRs lower than 1.0) among the subgroups in our stratified analyses (Figure 2).

Results from our sensitivity analyses can be found in the online supplementary material. The direction and magnitude of association between CRF and PRISm (Table S3), which is an alternative definition of pulmonary restriction, were similar to those found in the main analysis between CRF and RSP (Table 2), after adjusting for the covariates including BMI. Likewise, the significant associations between overweight and obesity with incident PRISm became non-significant when adjusting for CRF. While re-defining fitness and fatness using several alternative approaches resulted in a more heterogenous pattern of exposure-outcome relationships, the overall findings of these sensitivity analyses suggest that greater fitness paired with a leaner body habitus (e.g., normal weight, lower WC) is consistently associated with the lowest risk of RSP (Figures S1–S3), which aligns with the findings of our main joint analysis (Figure 1).

## DISCUSSION

We found that higher levels of CRF, independent of BMI, were associated with lower risks of RSP with a clear dose-response relationship among our five CRF categories. Obesity was significantly associated with incident RSP after adjusting for potential confounders, excluding CRF (Table 2). However, this association between obesity and RSP was no longer significant after further adjustment for CRF. Our joint analysis also indicated that high fitness, regardless of BMI group, was associated with a reduced risk of RSP compared with the ‘unfit and overweight/obese’ group. However, these results were attenuated when using alternative cut points for fitness (e.g., categorizing the lower third as ‘unfit’) or when separating the overweight and obese categories (Figures S2 and S3). Notably, there was no reduction in the risk of RSP among those classified as ‘unfit and normal weight,’ perhaps suggesting that leanness, in the absence of fitness, is not associated with RSP (Figure 1). The consistency of the association favoring higher CRF with reduced risk of RSP was also demonstrated among the various subgroups in our stratified analysis, including among different BMI categories (Figure 2).

A recent multi-center cohort study assessed the associations of self-reported physical activity on the incidence of RSP among 5,293 European men and women aged 36–82 years.[34] Results indicated that individuals who regularly engaged in vigorous physical activity (≥2 times/week with a cumulative duration ≥1 hour/week) had a 24% lower risk of developing RSP at follow-up compared to counterparts who were not vigorously active (adjusted relative risk, 0.76; 95% CI, 0.59–0.98]). These data can be considered analogous to the findings of the present study given that vigorous physical activity generally increases CRF.[34] We similarly found 22–41% lower risk of RSP among those with ‘high fitness’ (categories 2–5) compared to the lowest category of CRF (category 1).

Our findings suggest that low CRF is a potentially stronger risk factor for RSP than overweight or obesity. This finding is consistent with the broader ‘fat-but-fit paradigm’, which asserts that high CRF may attenuate disease risk associated with high body fatness.[14] Furthermore, being ‘unfit and normal weight’ may be insufficient to preserve health, which appears to be an increasingly accepted trait of the ‘fat-but-fit’ phenotype,[14] and is a notion supported by our joint CRF/BMI analyses (Figure 1). However, it is worth emphasizing that the lowest risks of RSP in our joint analysis and sensitivity analyses were seen in the ‘fit and normal weight’ group (Figure 1; supplementary figures S2 and S3), suggesting that maintaining a healthy body weight in addition to having high CRF is perhaps the most ‘ideal’ phenotype associated with reduced RSP risk.

Though no study has specifically assessed the prospective associations between CRF and RSP, a limited number of prospective studies have assessed the associations between CRF, lung function decline, and obstructive lung disease (e.g., COPD),[15, 16, 35] the latter of which is often comorbid with RSP.[36] These studies found CRF was associated with greater lung health even after adjusting for BMI. There is also evidence to suggest that physical activity, which generally improves CRF, can mitigate the age-related decline in general lung function (e.g., the loss of FEV_1_ or FVC over time),[37] which could delay the onset of RSP in later in life. We similarly found that lower levels of baseline CRF were associated with lower baseline FVC and a greater decline in FVC over time (Table S1), which might partially explain the greater risk of RSP among the ‘low fit’ phenotypes.

Although it was not examined in this study, one potential mechanism through which CRF could reduce the risk of RSP is inflammation.[38] Pulmonary restriction is partially driven by intrinsic inflammatory processes that damage the elastic properties of lung tissue, essentially restricting them from expanding properly.[6] High CRF is inversely associated with C-reactive protein (CRP), a biomarker of subclinical inflammation.[39, 40] High levels of CRP in young adulthood are linked to abnormal lung function in middle age,[41] and a recent prospective study of 3,332 adults postulated that high CRP among individuals with low CRF mediated the acceleration in lung function decline.[15] Another possible mechanism that explains the associations between CRF and incident RSP is the varied effect of fat distribution. Our second joint analysis (supplemental figure S1) showed slightly different associations with incident RSP when fitness and fatness were characterized by CRF and WC, relative to the associations observed in our first joint analysis (Figure 1). BMI alone does not accurately characterize the regional distribution of adiposity,[42] and abdominal obesity is strongly associated with lower static lung volumes (a hallmark feature of pulmonary restriction).[6] Low abdominal obesity alleviates mechanical compression of the lung and chest cavity,[6] which may partly explain the lower HRs for incident RSP observed in our second joint analyses. However, this assertation must be interpreted with caution because waist circumference data were only available for 70% of the 12,360 participants in the primary analytical sample.

### Limitations

A limitation of the present study is the observational design, which limits causal inference. There was a small yet significant correlation (r = 0.22, *P* < 0.001) between baseline CRF and baseline FVC, which may have increased the potential for reverse causation (i.e., RSP predictive of CRF). To minimize this, we excluded participants who developed RSP within one year of follow-up and also adjusted for baseline FVC. The method of participant entry into the ACLS cohort (i.e., employer/physician referral or self-referral), coupled with the monetary cost of undergoing health assessments, is also a limitation because it introduces selection bias. The ACLS consists mainly of non-Hispanic white, well-educated, and generally healthy and lean participants from mid-to-upper socioeconomic strata, which limits generalizability of our findings to this demographic group.[20] Strengths include our large sample size, prospective study design, the use of a maximal treadmill test to objectively assess CRF, the use of several stratified analyses and sensitivity analyses to assess the consistency of our findings, and the use of spirometry to objectively assess lung function.

### Conclusions

This study found a significant and inverse association between CRF and incident RSP, independent of BMI, which was consistent across the majority of subgroups within the sample. Our results demonstrated that the association between obesity and incident RSP was no longer significant after further adjustment for CRF. An evaluation of the joint associations of CRF and BMI indicated that fitness was associated with significantly lower risks of incident RSP, regardless of BMI, but the magnitude and significance of these associations were attenuated when alternative definitions of ‘fatness’ and ‘fitness’ were utilized, suggesting that the ‘fat-but-fit’ paradigm is potentially more nuanced in RSP compared to traditional cardiovascular disease outcomes. For instance, various indices of lung function (e.g., FVC) have been shown to be prospectively associated with cardiovascular disease, [1] so RSP may represent just one of the many pathways through which ‘fitness’ or ‘fatness’ influences cardiovascular health. Additional longitudinal studies utilizing racially and socio-economically diverse populations with a greater proportion of participants with overweight and obesity are needed to confirm our findings in the general adult population.

## Supplementary Material

Supp1

## Figures and Tables

**Table 1:** Participant and clinical characteristics at baseline

Characteristic	All	Cases	Non-cases	*P* value*
n (%)	12,360 (100)	900 (7.3)	11,460 (92.7)	-
Age, mean (SD), y	44.1 (9.3)	43.4 (8.9)	44.1 (9.3)	0.042
Women, No. (%)	2042 (16.5)	122 (13.6)	1920 (16.8)	0.013
Height, mean (SD), cm	176.7 (8.4)	177.1 (7.9)	176.6 (8.4)	0.100
BMI, mean (SD), kg/m^2^	25.3 (3.5)	25.6 (3.9)	25.3 (3.4)	0.004
BMI category, No. (%)				0.043
Normal weight	6119 (49.5)	430 (47.8)	5689 (49.6)	
Overweight	5172 (41.8)	372 (41.3)	4800 (41.9)	
Obese	1069 (8.7)	98 (10.9)	971 (8.5)	
Smoking status, No. (%)				<0.001
Never	6222 (50.3)	403 (44.8)	5819 (50.8)	
Former	4311 (34.9)	314 (34.9)	3997 (34.9)	
Current	1872 (14.8)	183 (20.3)	1644 (14.4)	
Heavy alcohol drinking†, No. (%)	2370 (19.2)	171 (19.0)	2199 (19.2)	0.890
Treadmill duration, mean (SD), mins	18.0 (4.9)	16.7 (4.8)	18.1 (4.9)	<0.001
Maximal METs achieved on treadmill, mean (SD)	11.7 (2.4)	11.1 (2.3)	11.7 (2.4)	<0.001
Meets aerobic physical activity guidelines‡, No. (%)	5125 (41.5)	285 (31.7)	4840 (42.2)	<0.001
Total weekly physical activity (MET mins), mean (SD)	714.5 (1116.3	522.6 (913.4)	729.6 (1129.4)	<0.001
FEV_1_ % of predicted, mean (SD)	95.1 (12.3)	85.3 (11.1)	95.8 (12.1)	<0.001
FVC % of predicted, mean (SD)	97.0 (10.7)	87.2 (8.7)	97.8 (10.4)	<0.001
FVC, mean (SD), L	4.8 (0.9)	4.4 (0.8)	4.8 (0.9)	<0.001
Annual change in FVC, mean (SD), L	−0.03 (0.2)	−0.3 (0.3)	−0.02 (0.2)	<0.001
FEV_1_/FVC %, mean (SD)	78.5 (0.1)	78.3 (0.1)	78.5 (0.1)	0.374
Diabetes, No. (%)	617 (5.0)	71 (7.9)	546 (4.8)	<0.001
Hypertension, No. (%)	7306 (59.1)	594 (66)	6712 (58.6)	<0.001

Abbreviations: BMI, body mass index; FEV1, forced expiratory volume in 1-second; FEV1/FVC, the ratio between FEV1 and FVC; FVC, forced vital capacity; MET, metabolic equivalent; No., number; SD, standard deviation;

* *P*-value for the comparison between cases and non-cases: χ2 (categorical) or general linear models (continuous).

† Heavy drinking defined as >7 alcoholic drinks/week for women, and >14 alcoholic drinks/week for men.

‡ Meeting aerobic physical activity guidelines is defined as ≥500 MET-min/week.

**Table 2:** Hazard ratios (HRs) and 95% confidence intervals (CIs) for the associations of cardiorespiratory fitness (CRF) and body mass index (BMI) with restrictive spirometry pattern (RSP)

			**HR (95% CI)**		
**CRF categories** *	**Cases(%)**	**n (%)**	**Model 1**	**Model 2**	**Model 3**

**1 (Least fit)**	184 (14.2)	1298 (10.5)	1.00 [Reference]	1.00 [Reference]	1.00 [Reference]
**2**	191 (9.0)	2129 (17.2)	**0.61 (0.50–0.74)**	**0.75 (0.61–0.92)**	**0.78 (0.63–0.96)**
**3**	169 (7.0)	2408 (19.5)	**0.46 (0.38–0.57)**	**0.65 (0.53–0.81)**	**0.68 (0.54–0.86)**
**4**	199 (6.3)	3170 (25.6)	**0.41 (0.34–0.50)**	**0.66 (0.53–0.82)**	**0.70 (0.55–0.88)**
**5 (Most fit)**	157 (4.7)	3355 (27.1)	**0.30 (0.24–0.37)**	**0.54 (0.42–0.70)**	**0.59 (0.45–0.77)**
***P* for linear trend**			**<0.001**	**<0.001**	**0.0003**
**Per 1 MET increase in CRF**			**0.83 (0.80–0.86)**	**0.92 (0.89–0.96)**	**0.93 (0.90–0.97)**

			**HR (95% CI)**		
**BMI** ^†^	**Cases (%)**	**n (%)**	**Model 1**	**Model 2**	**Model 3**

**Normal weight**	430 (7.0)	6119 (49.5)	1.00 [Reference]	1.00 [Reference]	1.00 [Reference]
**Overweight**	372 (7.2)	5172 (41.8)	**1.22 (1.06–1.42)**	1.06 (0.91–1.23)	0.98 (0.84–1.14)
**Obesity**	98 (9.2)	1069 (8.6)	**1.90 (1.52–2.38)**	**1.30 (1.03–1.64)**	1.08 (0.84–1.39)
***P* for linear trend**			**<0.001**	0.055	0.765
**Per 1 unit increase in BMI**			**1.07 (1.05–1.09)**	**1.04 (1.01–1.06)**	1.02 (0.996–1.04)

Abbreviations: MET, metabolic equivalent.

Model 1 was adjusted for sex, age (years), and examination year.

Model 2 was adjusted for Model 1 plus smoking status (never, former, current), heavy alcoholic intake (yes or no), meeting the aerobic physical activity guidelines (yes or no), diabetes (yes or no), hypertension (yes or no), and baseline FVC (L).

Model 3 was adjusted for Model 2 plus BMI (kg/m2) in the CRF analysis, or CRF (METs) in the BMI analysis.

* Categories of CRF were based on of the age and sex distribution of CRF for the entire Aerobics Center Longitudinal Study (ACLS) cohort.

† Normal weight (BMI <25.0 kg/m^2^), overweight (BMI 25.0–29.9 kg/m^2^), and obesity (BMI ≥30.0 kg/m^2^).

## Data Availability

Data is not publicly available but may be obtained upon reasonable request.

## SUMMARY BOX

### What is already known on this topic:

- Restrictive spirometry pattern (RSP) is a prevalent form of respiratory impairment that significantly increases the risk of premature mortality compared to normal (healthy) spirometry.
- The development of RSP is closely linked with body mass index (BMI), with overweight and obesity being strong risk factors.

### What this study adds:

- High cardiorespiratory fitness (CRF) was associated with a lower risk of RSP, independent of BMI.
- The associations between overweight or obesity and incident RSP became non-significant when adjusting for CRF.

### How this study might affect research, practice, or policy:

- These findings suggest that higher CRF is associated with a reduced risk of RSP, regardless of BMI. Physical activity, which can increase CRF, should be considered in future public health studies as it may promote lung health alone or in combination with standard weight-related guidance.
