# Supplementary material for: Associations of Cardiorespiratory Fitness and Body Mass Index with Incident Restrictive Spirometry Pattern
Source: Br J Sports Med. Author manuscript; Available in PMC 2023 Aug 5. (PMC10323034; doi:10.1136/bjsports-2022-106136)
Supplement: Supp1 [file NIHMS1867175-supplement-Supp1.pdf]

Supplementary material

**Table S1** Participant and clinical characteristics at baseline by categories of cardiorespiratory fitness						
**Characteristic**	**Category 1 (Least fit)**	**Category 2**	**Category 3**	**Category 4**	**Category 5 (Most fit)**	**P-value**
n (%)	1298 (10.5)	2129 (17.2)	2408 (19.5)	3170 (25.6)	3355 (27.1)	-
Age, mean (SD), y	43.1 (8.4)	43.1 (8.8)	44.3 (9.1)	44.2 (9.3)	44.7 (9.9)	<0.001
Women, No.(%)	149	302	333	578	680	<0.001
Height, mean (SD), cm	176.6 (7.9)	176.7 (8.2)	177.2 (8.2)	176.7 (8.6)	176.3 (8.7)	0.001
BMI, mean (SD), kg/m^2	28.1 (4.6)	26.3 (3.5)	25.7 (3.2)	24.9 (2.9)	23.8 (2.5)	<0.001
BMI category, No.(%)						
Normal weight	339 (26.1)	774 (36.4)	1011 (42.0)	1666 (52.6)	2329 (69.4)	
Overweight	587 (45.2)	1060 (49.8)	1166 (48.4)	1372 (43.3)	987 (29.4)	<0.001
Obese	372 (28.7)	295 (13.9)	231 (9.6)	132 (4.2)	39 (1.2)	
Smoking status, No.(%)						
Never	492 (37.9)	947 (44.5)	1187 (49.3)	1703 (53.7)	1893 (56.4)	
Former	446 (34.4)	737 (34.6)	829 (34.4)	1098 (34.6)	1201 (35.8)	<0.001
Current	360 (27.7)	445 (20.9)	392 (16.3)	369 (11.6)	261 (7.8)	
Heavy alcohol drinking†, No. (%)	266 (20.5)	389 (18.3)	486 (20.2)	607 (19.2)	622 (18.5)	0.284
Treadmill duration, mean (SD), mins	11.3 (2.7)	14.4 (2.6)	16.6 (2.7)	19.0 (3.1)	23.1 (3.8)	<0.001
Maximal METs achieved on treadmill, mean (SD)	8.6 (1.2)	9.9 (1.2)	11.0 (1.2)	12.1 (1.4)	14.2 (2.1)	<0.001
Meets aerobic physical activity guidelines‡, No. (%)	100 (7.7)	322 (15.1)	669 (27.8)	1549 (48.9)	2485 (74.1)	<0.001
Total weekly physical activity (MET mins), mean (SD)	110.5 (392.6)	210.0 (473.2)	405.7 (926.6)	714.2 (876.0)	1490.2 (1440.9)	<0.001
FEV_1 % of predicted, mean (SD)	90.7 (12.6)	92.9 (12.3)	94.5 (12.1)	96.1 (11.9)	97.6 (12.1)	<0.001
FVC % of predicted, mean (SD)	92.8 (10.0)	94.9 (10.3)	96.1 (10.5)	97.8 (10.5)	99.8 (10.5)	<0.001
FVC, mean (SD), L	4.6 (0.8)	4.7 (0.8)	4.8 (0.9)	4.8 (0.9)	4.9 (1.0)	0.039
Annual change in FVC, mean (SD), L	-0.05 (0.2)	-0.04 (0.2)	-0.03 (0.2)	-0.03 (0.2)	-0.03 (0.1)	<0.001
FEV_1/FVC %, mean (SD)	78.3 (0.1)	78.6 (0.1)	78.6 (0.1)	78.6 (0.1)	78.3 (0.1)	0.243
Diabetes, No. (%)	110 (8.5)	146 (6.9)	124 (5.2)	141 (4.5)	96 (2.9)	<0.001
Hypertension, No. (%)	918 (70.7)	1382 (64.9)	1435 (59.6)	1823 (57.5)	1748 (52.1)	<0.001

Abbreviations: BMI, body mass index; FEV1, forced expiratory volume in 1-second; FEV1/FVC, the ratio between FEV1 and FVC; FVC, forced vital capacity; MET, metabolic equivalent; No., number; SD, standard deviation;
**P*-value for the comparisons across categories of CRF: χ2 (categorical variables) or general linear models (continuous variables).
†Heavy drinking defined as >7 alcoholic drinks/week for women, and >14 alcoholic drinks/week for men.
‡Meeting aerobic physical activity guidelines is defined as ≥500 MET-min/week.

**Table S2** Participant and clinical characteristics at baseline by categories of body mass index				
**Characteristic**	**Obese** (BMI ≥30kg/m^2)	**Overweight** (BMI: 25 to 29.9kg/m^2)	**Normal weight** (BMI <25kg/m^2)	***P* value**[*]
n (%)	1069 (8.6)	5172 (41.8)	6119 (49.5)	-
Age, mean (SD), y	44.6 (8.5)	45.0 (8.9)	43.2 (9.6)	<0.001
Women, No. (%)	85 (8.0)	278 (5.38)	1679 (27.4)	<0.001
Height, mean (SD), cm	177.5 (8.3)	178.2 (7.3)	175.2 9.0)	<0.001
BMI, mean (SD), kg/m^2	32.6 (2.8)	26.9 (1.3)	22.7 (1.7)	<0.001
Smoking status, No. (%)				
Never	516 (48.3)	2485 (48.1)	3221 (52.6)	
Former	346 (32.4)	1824 (35.3)	2141 (35.0)	<0.001
Current	207 (19.4)	863 (16.7)	757 (12.4)	
Heavy alcohol drinking†, No. (%)	217 (20.3)	993 (19.2)	1160 (19.0)	0.588
Treadmill duration, mean (SD), mins	14.4 (3.9)	17.6 (4.4)	19.0 (5.2)	<0.001
Maximal METs achieved on treadmill, mean (SD)	10.0 (1.8)	11.5 (2.1)	12.2 (2.6)	<0.001
Meets aerobic physical activity guidelines‡, No. (%)	358 (33.5)	2004 (38.8)	2763 (45.2)	<0.001
Total weekly physical activity (MET mins), mean (SD)	521.4 (896.0)	621.7 (952.0)	826.7 (1258.9)	<0.001
FEV_1 % of predicted, mean (SD)	94.5 (13.2)	95.5 (12.3)	94.8 (12.2)	0.306
FVC % of predicted, mean (SD)	94.8 (11.4)	97.0 (10.7)	97.4 (10.5)	<0.001
FVC, mean (SD), L	4.8 (0.8)	5.0 (0.8)	4.7 (1.0)	<0.001
Annual change in FVC, mean (SD), L	-0.02 (0.2)	-0.03 (0.2)	-0.03 (0.2)	0.246
FEV_1/FVC %, mean (SD)	79.6 (11.4)	78.5 (0.1)	78.3 (0.1)	<0.001
Diabetes, No. (%)	97 (9.1)	259 (5.0)	261 (4.3)	<0.001
Hypertension, No. (%)	863 (80.7)	3450 (66.7)	2993 (48.9)	<0.001

Abbreviations: BMI, body mass index; FEV1, forced expiratory volume in 1-second; FEV1/FVC, the ratio between FEV1 and FVC; FVC, forced vital capacity; MET, metabolic equivalent; No., number; SD, standard deviation;

**P*-value for the comparison between cases and non-cases: $\chi 2$ (categorical) or general linear models (continuous).

†Heavy drinking defined as >7 alcoholic drinks/week for women, and >14 alcoholic drinks/week for men.

‡Meeting aerobic physical activity guidelines is defined as ≥500 MET-min/week.

Table S3 Hazard ratios (HRs) and 95% confidence intervals (CIs) for the associations of cardiorespiratory fitness (CRF) and body mass index (BMI) on Preserved Ratio Impaired Spirometry (PRISm)

			HR (95% CI)		
**CRF categories***	**Cases (%)**	**n (%)**	**Model 1**	**Model 2**	**Model 3**
**1 (Least fit)**	187 (14.6)	1277 (10.5)	1.00 [Reference]	1.00 [Reference]	1.00 [Reference]
**2**	244 (11.8)	2077 (17.1)	**0.76 (0.64-0.94)**	**0.80 (0.66-0.97)**	**0.82 (0.67-0.99)**
**3**	221 (9.4)	2356 (19.4)	**0.60 (0.49-0.73)**	**0.63 (0.52-0.77)**	**0.65 (0.53-0.80)**
**4**	223 (7.2)	3111 (25.7)	**0.45 (0.37-0.54)**	**0.46 (0.37-0.67)**	**0.48 (0.39-0.60)**
**5 (Most fit)**	205 (6.2)	3301 (27.2)	**0.37 (0.31-0.46)**	**0.39 (0.31-0.49)**	**0.41 (0.32-0.52)**
***P* for trend**			**<0.001**	**<0.001**	**<0.001**
**Per 1 MET increase in CRF**			**0.84 (0.82-0.87)**	**0.84 (0.81-0.87)**	**0.85 (0.82-0.88)**

			HR (95% CI)		
**BMI†**	**Cases (%)**	**N (%)**	**Model 1**	**Model 2**	**Model 3**
**Normal weight**	526 (8.8)	5973 (49.3)	1.00 [Reference]	1.00 [Reference]	1.00 [Reference]
**Overweight**	443 (8.7)	5080 (41.9)	**1.25 (1.09-1.43)**	**1.18 (1.03-1.35)**	1.00 (0.88-1.16)
**Obesity**	111 (10.4)	1069 (8.8)	**1.83 (1.48-2.25)**	**1.60 (1.29-1.98)**	1.08 (0.86-1.36)
***P* for trend**			**<.0001**	**<.0001**	0.621
**Per 1 unit increase in BMI**			**1.06 (1.04-1.08)**	**1.05 (1.03-1.07)**	1.01 (0.99-1.03)

Abbreviations: MET, metabolic equivalent.
Model 1 was adjusted for sex, age (years), and examination year.
Model 2 was adjusted for Model 1 plus smoking status (never, former, current), heavy alcoholic intake (yes or no), meeting the aerobic physical activity guidelines (yes or no), diabetes (yes or no), hypertension (yes or no), and baseline FVC (L).
Model 3 was adjusted for Model 2 plus BMI (kg/m2) in the CRF analysis, or CRF (METs) in the BMI analysis.
*Categories of CRF were based on of the age and sex distribution of CRF for the entire Aerobics Center Longitudinal Study (ACLS) cohort.
†Normal weight (BMI <25.0 kg/m^2), overweight (BMI 25.0-29.9 kg/m^2), and obesity (BMI ≥30.0 kg/m^2).

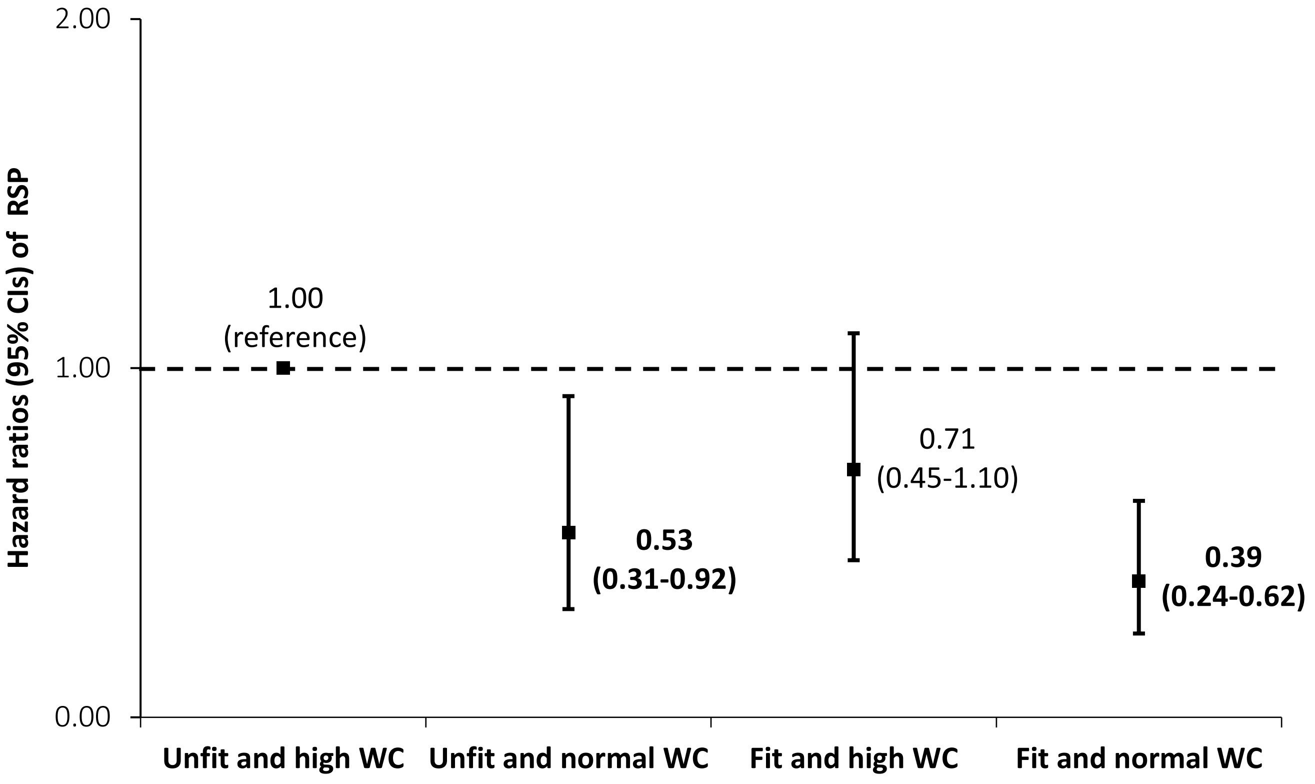

Figure S1. Joint associations of cardiorespiratory fitness (CRF) and waist circumference (WC) with incident restrictive spirometry pattern (RSP).

Participants were divided into four groups based on combined categories of cardiorespiratory fitness (unfit or fit) and waist circumference (WC) (high WC or normal WC). 'Unfit' was defined as the lower 20% of the cardiorespiratory fitness distribution, and 'fit' was defined as the upper 80%. High WC was defined as >102cm and >88cm for men and women, respectively. Normal WC was defined as ≤102cm and ≤88cm for men and women, respectively. The cox proportional hazard model was adjusted for sex, age (years), examination year, smoking status (never, former, current), heavy alcohol intake (yes or no), meeting the aerobic physical activity guidelines (yes or no), diabetes (yes or no), hypertension (yes or no), body mass index (BMI; kg/m^2), and baseline FVC (L). The number of participants (and cases of RSP) in the 'Unfit and high WC', 'Unfit and normal WC', 'fit and high WC', and 'Fit and normal WC' groups 275 (35), 359 (34), 797 (51), and 7254 (326), respectively.

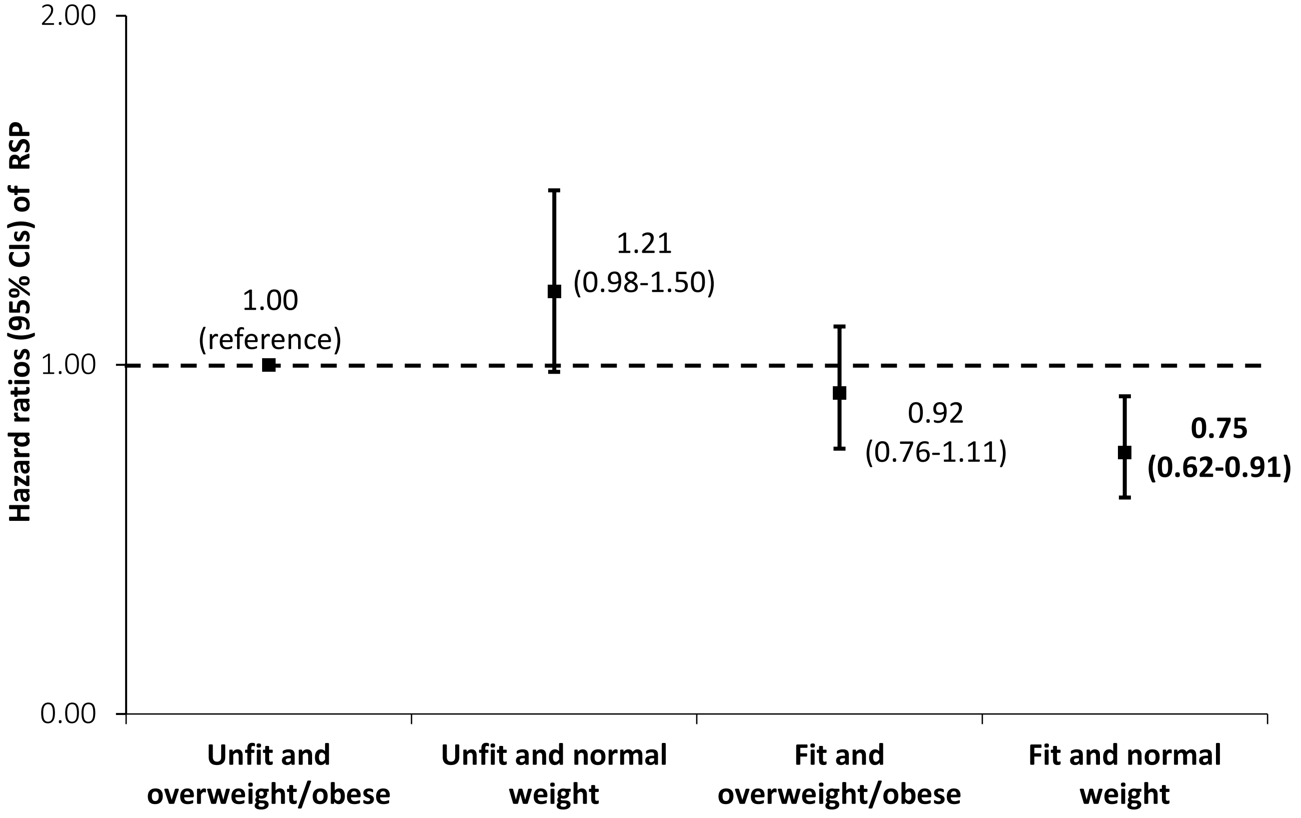

Figure S2. Joint associations of cardiorespiratory fitness (CRF) and body mass index (BMI) categories with incident restrictive spirometry pattern (RSP).

Participants were divided into four groups based on combined categories of cardiorespiratory fitness ('unfit' or 'fit') and body mass index (normal weight or overweight/obesity). 'Unfit' was defined as the **bottom third** of the cardiorespiratory fitness distribution, and 'fit' was defined as the **upper two-thirds**. Normal weight was defined as body mass index <25.0 kg/m2, while overweight/obese was defined as was ≥25 kg/m2. The cox proportion hazard model was adjusted for sex, age (years), examination year, smoking status (never, former, current), heavy alcohol intake (yes or no), meeting the aerobic physical activity guidelines (yes or no), diabetes (yes or no), hypertension (yes or no), and baseline FVC (L). The number of participants (and cases of restrictive spirometry pattern) in the 'unfit and overweight/obese', 'unfit and normal weight', 'fit and overweight/obese', and 'fit and normal weight' categories were 2555 (246), 1349 (157), 3686 (224) and 4770 (273) respectively. Bolded values indicate statistically significant difference from the reference group (P <0.05).

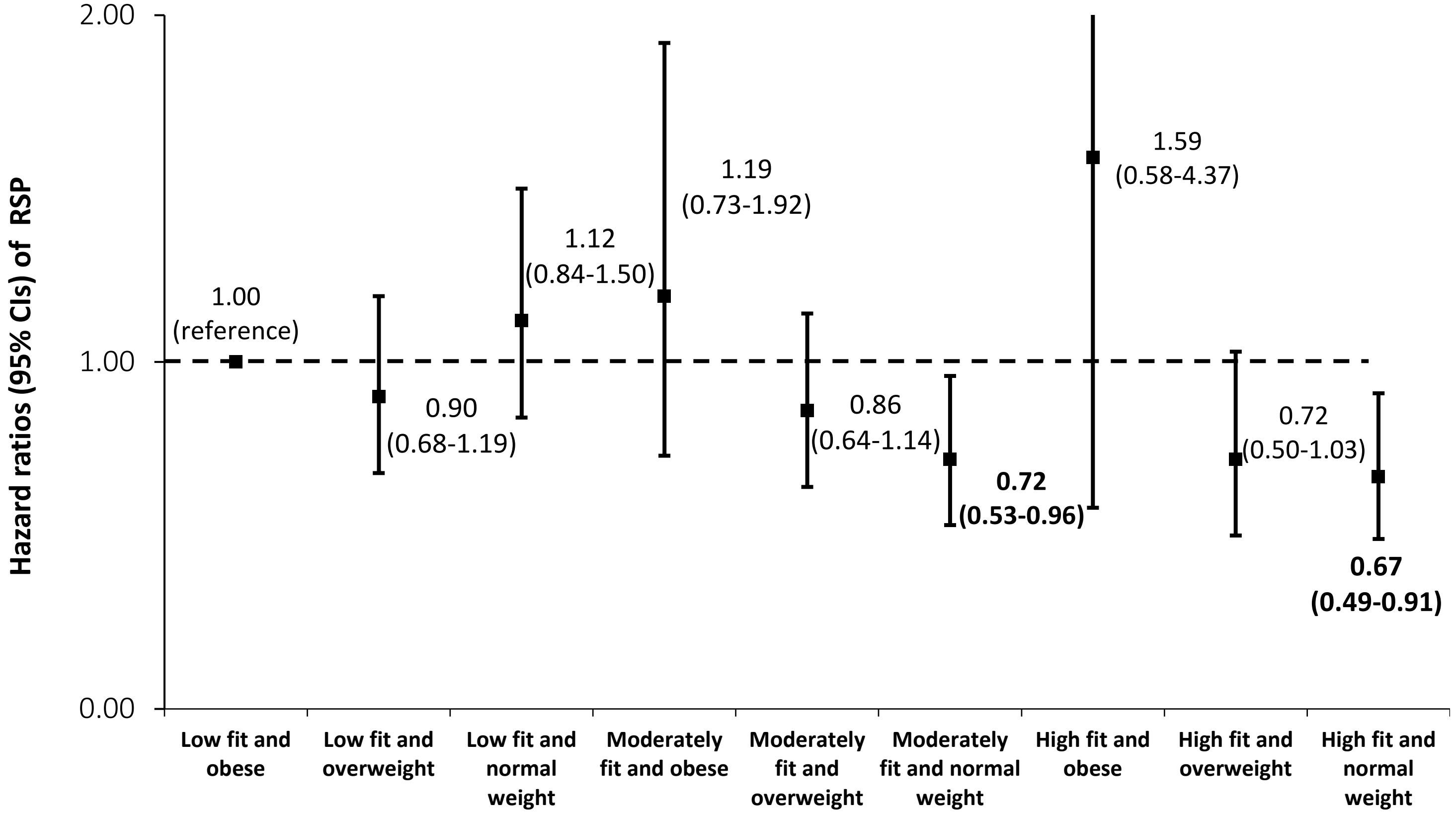

Figure S3. Joint associations of cardiorespiratory fitness (CRF) and body mass index (BMI) with incident restrictive spirometry pattern (RSP).

Participants were divided into nine groups based on combined categories of cardiorespiratory fitness (‘low fit’, ‘moderately fit’, ‘high fit’) and body mass index (normal weight, overweight, obese). ‘Low fit’ was defined as the **bottom third** (Tertile 1) of the cardiorespiratory fitness distribution, ‘moderately fit’ was defined as the **middle third** (Tertile 2), and ‘high fit’ was defined as the **upper third** (Tertile 3). Normal weight, overweight, and obese were defined as body mass index (BMI) <25.0 kg/m^2, 25.0-29.9kg/m^2, and ≥30kg/m^2, respectively. The cox proportion hazard model was adjusted for sex, age (years), examination year, smoking status (never, former, current), heavy alcohol intake (yes or no), meeting the aerobic physical activity guidelines (yes or no), diabetes (yes or no), hypertension (yes or no), and baseline FVC (L). The number of participants (and cases of restrictive spirometry pattern) in the ‘low fit and obese’, ‘low fit and overweight, ‘low fit and normal weight’, ‘moderately fit and obese’, ‘moderately fit and overweight’, ‘moderately fit and normal weight’, ‘high fit and obese’, ‘high fit and overweight’, and ‘high fit and normal weight’ are 724 (72), 1831 (174), 1349 (157), 290 (22), 2089 (140), 2064 (137), 55 (4), 1252 (58), and 2706 (136) respectively. Bolded values indicate statistically significant difference from the reference group (P <0.05).
